# P10 Biogenic silver and gold nanoparticles from *M. esculenta:* combating pandrug-resistant from the clinical isolates of patients with urinary tract infections

**DOI:** 10.1093/jacamr/dlag102.016

**Published:** 2026-06-26

**Authors:** Pratima Gupta, Diksha Rani, U C Banerjee, Deepjyoti Kalita

**Affiliations:** All India Institute of Medical Sciences, Rishikesh, 249203, Uttarakhand, India; State VRDL All India Institute of Medical Sciences, Rishikesh, 249203, Uttarakhand, India; MIT University Mohali, India; All India Institute of Medical Sciences, Guwahati, India

## Abstract

**Background:**

Pan-drug-resistant bacterial infections are a serious threat to public health since pathogenic strains are resistant to a variety of antimicrobials. In the field of nanotechnology, nanoparticles(NPs) serve as an excellent antibacterial agent. The current study synthesized silver and gold nanoparticles using the leaf of *M. esculenta* and analysed its antibacterial activity against pan-resistant strains of urinary tract infection.

**Objective:**

To synthesize silver and gold nanoparticles using leaf extract of *M. esculenta* and evaluate their antibacterial efficacy against pan-drug-resistant clinical isolates obtained from patients with urinary tract infections.

**Methods:**

The pan-resistant strain of UTI was identified using the Kirby-Bauer disc diffusion method. Plant extract was prepared followed by silver and gold NPs synthesis. These NPs were characterized through UV-Vis, Transmission electron microscopy (TEM), FTIR, and XRD. Antibacterial activity of these NPs was seen through disc diffusion and MIC.

**Results:**

Results of characterization showed that biosynthesized NPs were spherical in shape, consisted of various functions such as phenols, alkene, and alkyne, etc. XRD results showed that biosynthesized NPs were crystalline in nature. These silver and gold NPs showed excellent results against pan-resistant strains against *E. coli, K. pneumoniae, P. aeruginosa, P. vulgaris, A. baumannii S. aureus,* and *E. faecalis.* Disc diffusion assay showed an increase in the zone of inhibition with increasing NPs concentrations. MIC of silver NPs against *E. coli*, *K. pneumoniae,* and *A. baumannii* was achieved at 50 mg/L, whereas 75 mg/L MIC was achieved against *P. aeruginosa, S. aureus, P. vulgaris* and *E. faecalis.* MIC of gold NPs was achieved at 200 mg/L against E.coli, *K. pneumoniae*, *A. baumannii, P. aeruginosa,* and *P. vulgaris* whereas 100 mg/L was achieved against *S. aureus* and *E. faecalis*. One way ANOVA test showed a significant *P* value (<0.001) in the mean differences between the MIC of pathogens.

**Conclusions:**

The current findings showed considerable antibacterial activity at low concentrations. To the best of our knowledge, this is the first study that uses the leaves of *M. esculenta* for silver and gold NPs synthesis and tested their antibacterial activity against pan-resistant resistant (clinical isolates) uropathogenic strains.

